# Pre-Trained Joint Model for Intent Classification and Slot Filling with Semantic Feature Fusion

**DOI:** 10.3390/s23052848

**Published:** 2023-03-06

**Authors:** Yan Chen, Zhenghang Luo

**Affiliations:** 1 School of Computer and Electronic Information, Guangxi University, Nanning 530004, China; 2 Guangxi Key Laboratory of Multimedia Communication and Network Technology, Guangxi University, Nanning 530004, China; 3 Guangxi Intelligent Digital Services Research Center of Engineering Technology, Nanning 530004, China

**Keywords:** spoken language understanding, intent classification, slot filling, joint model

## Abstract

The comprehension of spoken language is a crucial aspect of dialogue systems, encompassing two fundamental tasks: intent classification and slot filling. Currently, the joint modeling approach for these two tasks has emerged as the dominant method in spoken language understanding modeling. However, the existing joint models have limitations in terms of their relevancy and utilization of contextual semantic features between the multiple tasks. To address these limitations, a joint model based on BERT and semantic fusion (JMBSF) is proposed. The model employs pre-trained BERT to extract semantic features and utilizes semantic fusion to associate and integrate this information. The results of experiments on two benchmark datasets, ATIS and Snips, in spoken language comprehension demonstrate that the proposed JMBSF model attains 98.80% and 99.71% intent classification accuracy, 98.25% and 97.24% slot-filling F1-score, and 93.40% and 93.57% sentence accuracy, respectively. These results reveal a significant improvement compared to other joint models. Furthermore, comprehensive ablation studies affirm the effectiveness of each component in the design of JMBSF.

## 1. Introduction

The advancement of Artificial Intelligence (AI) technology has resulted in the proliferation of intelligent voice assistants in everyday life. Spoken Language Understanding (SLU) is a crucial aspect of these systems, converting users’ inputs in the form of speech or text into structured semantic features that can be interpreted by the machine. As such, an efficient SLU approach is indispensable in accurately comprehending users’ requirements [1,2]. SLU encompasses two subtasks, namely, intent classification and slot filling. Intent classification aims to identify the specific purpose behind a user’s utterance [3]. Compared to traditional machine learning methods [4] and rule-based methods [5] for intent classification, the use of deep learning models provides a more accurate classification of semantic features [6] by effectively capturing the contextual relationships between utterances and dependencies between remote word elements.

Slot filling, on the other hand, is a sequential annotation task that extracts structured information from sentences and assigns semantic tags to lexical elements in predefined semantic slots. This is accomplished by labeling the lexical elements in a sentence with the BIO (Begin/Inside/Outside) format, where “B” represents the beginning of an entity fragment, “I” signifies the middle of an entity fragment, and “O” indicates that the character does not belong to any entity. The word element is labeled as a “B-X”, “I-X,” or “O” semantic tag, with “B-X” denoting the beginning of semantic slot X, “I-X” representing a non-beginning element of semantic slot X, and “O” signifying that the word element does not belong to any semantic slot. X can consist of a single word or multiple words connected by “-” [7]. As illustrated in Table 1, a sentence sample “add this track to new hip hop” would have the lexical element “track” labeled as “B-music_item”, the word “new” labeled as “B-playlist”, and the words “hip” and “hop” labeled as “I-playlist”. The sentence is then classified as “AddToPlaylist” for intent classification.

During early NLU research, pipeline methods were used to model two subtasks separately, which failed to consider their correlation and hindered effective problem-solving. Subsequent research has shifted towards joint modeling to better address this issue [8,9,10]. However, previous joint models often underperformed at the sentence level due to their underutilization of semantic features and lack of correlation between them. To address these problems, this paper proposes a semantic feature fusion layer to explicitly correlate the unused semantic features in the model and better utilize overall semantic features, resulting in improved sentence accuracy. The proposed model uses pre-trained BERT to extract semantic features and the semantic feature fusion layer to fuse multi-layer contextual semantic features.

This paper’s main contributions are as follows:

(1). Proposing an effective semantic feature fusion layer that improves the utilization of semantic features and mutual information between tasks, leading to better sentence-level accuracy than other models;
(2). Introducing the Joint Model based on BERT with Semantic Fusion (JMBSF), which employs the pre-trained BERT and semantic fusion layer to combine multi-layer contextual semantic features extracted by BERT;
(3). Demonstrating the effectiveness of the proposed model structure and identifying the optimal value for the semantic feature fusion layer parameter K through ablation experiments;
(4). Achieving state-of-the-art performance in two public datasets, especially in terms of sentence-level accuracy.

## 2. Related Work

In the field of SLU, previous approaches have utilized pipelined models that treat intent classification and slot filling as separate tasks [11], disregarding their interdependence. To address this issue, researchers have proposed joint models based on parallel training. Liu et al. [12] employed a Recurrent Neural Network (RNN) model to jointly extract semantic features. Wang et al. [13] proposed a bi-directional association model using bi-directional long and short-term memory networks (Bi-LSTM) and a bi-directional association gating layer. Lixian et al. [14] introduced a Bi-LSTM model with a slot-gated and a Conditional Random Field (CRF) structure, incorporating an attention mechanism in dual-task training. Another joint modeling approach involves extracting semantic features from a large corpus using pre-trained models, followed by fine-tuning with a smaller annotated corpus [15,16]. Chen et al. [2] introduced the pre-training model BERT into their joint model, which utilizes the BERT model for intent classification and fine-tunes the two tasks using cross-entropy loss to maximize the conditional probability $P\left(y^{intent},y^{\left(slots|x\right)}\right)$. Qin et al. [17] introduced the pre-training model BERT with Stack-Propagation, proposing a novel framework for SLU that better incorporates intent information into slot filling through the utilization of a joint model with Stack-Propagation and token-level intent classification to capture intent semantic knowledge and alleviate error propagation. Pang et al. [18] introduced the pre-training model BERT with a typed abstraction mechanism, which presents a novel approach to enhance the performance of intent classification by leveraging encoded information from semantic feature tasks and using a typed abstraction mechanism. Their typed iteration approach further facilitates the bi-directional integration of encoded information, reducing the detrimental impact of error propagation. Despite the widespread application of pre-trained models in joint modeling, the BERT model’s use of the coded output from the last layer as input for the downstream task leads to ignoring the correlation between semantic feature vectors, thereby not fully utilizing the interrelationship of contextual semantic feature information between the two tasks.

In this paper, a Joint Model based on BERT with Semantic Fusion (JMBSF) is presented to overcome the limitations of previous approaches in the joint modeling of SLU. The JMBSF model utilizes the pre-trained BERT model to extract semantic features and incorporates the contextual semantic features from multiple layers through semantic fusion. Furthermore, the model incorporates the bi-directional contextual information with the use of a Bi-LSTM structure and fuses semantic features in the intent classification sub-task. Additionally, the CRF module is used in the slot-filling sub-task to capture the temporal dependencies in the labels. The model is optimized using the focal loss [19] and LazyAdam optimizer [20]. The proposed JMBSF model is evaluated on ATIS [21] and Snips [22] datasets and demonstrates improved performance compared to the baseline models.

## 3. Methodology

**Figure 1:**
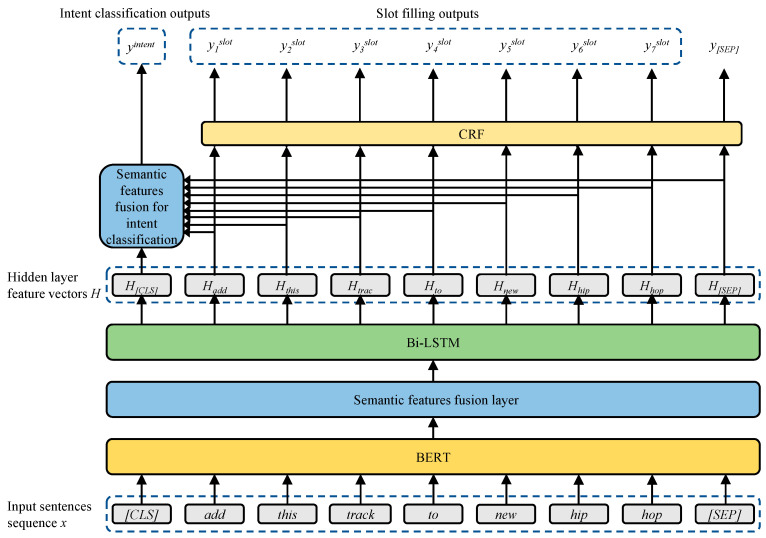
Model architecture.

The upstream encoder component of the proposed JMBSF model consists of three elements: the BERT layer; the semantic feature fusion layer; and the Bi-LSTM layer. As illustrated in Figure 1, the model first employs BERT to encode and semantically extract the input sentence sequence. It then fuses the multi-layer semantic feature vectors obtained from the BERT encoding with semantic features, before accessing the Bi-LSTM to produce more efficient bi-directional contextual location encoding information. In the two downstream tasks, the JMBSF integrates the intent features with the slot features for fusion and uses the fused information to derive the sentence-level classification results required for the intent classification task. It passes the slot features into the CRF layer to attain the word-level classification results necessary for the slot-filling task.

### 3.1. BERT

BERT, proposed by Devlin et al. [15], has been extensively employed in NLP tasks, achieving remarkable results. The JMBSF model in this paper employs BERT as the upstream semantic feature extractor. BERT is comprised of a stacked encoder architecture of multiple transformers [23], where the input layer is a combination of WordPiece based [24] word embedding, positional embedding, and segment-marker embedding. The input layer processes the input sentences to produce an encoding sequence. BERT also adds special word meta-tokens to the beginning and end of sentences. Given a token sequence $x=\left(x_{1},x_{2},x_{3},x_{4},\cdots x_{n-1},x_{n}\right)$, where $x_{1}$ is the [CLS] token and $x_{n}$ is the [SEP] token, BERT outputs a word-embedding sequence vector $h=\left(h_{1},h_{2},h_{3},h_{4},\cdots h_{n-1},h_{n}\right)$.

The multi-layer encoder stack of BERT effectively extracts semantic features in text sequences, utilizing multi-headed self-attentive, Layernorm, residual-connected, and fully-connected layers in each encoder. BERT is pre-trained on a large corpus of unlabeled data, allowing it to learn the structured semantic features in the corpus [2]. As a result, BERT is highly capable of encoding contextual utterances and can be fine-tuned for various NLP tasks [25,26]. The generalization capability of the model is significantly improved by its pre-training on the large corpus.

### 3.2. The BERT-Semantic Feature Fusion Layer

The BERT employs a serial arrangement of multiple encoding layers to extract semantic features. Each layer of the encoder generates specific semantic features that are subsequently input to the subsequent layer for re-encoding. The final output, represented by $H=\left(H_{1},H_{2},H_{3},H_{4},\cdots H_{n-1},H_{n}\right)$, generated by the last layer of the encoder is conveyed to the downstream task [15].

The semantic feature vectors generated by each encoding layer of BERT possess contextual semantic features, and the fusion of these feature vectors prior to feeding them to the downstream task results in more efficient utilization of the semantic features compared to using the output of the last layer alone.

**Figure 2:**
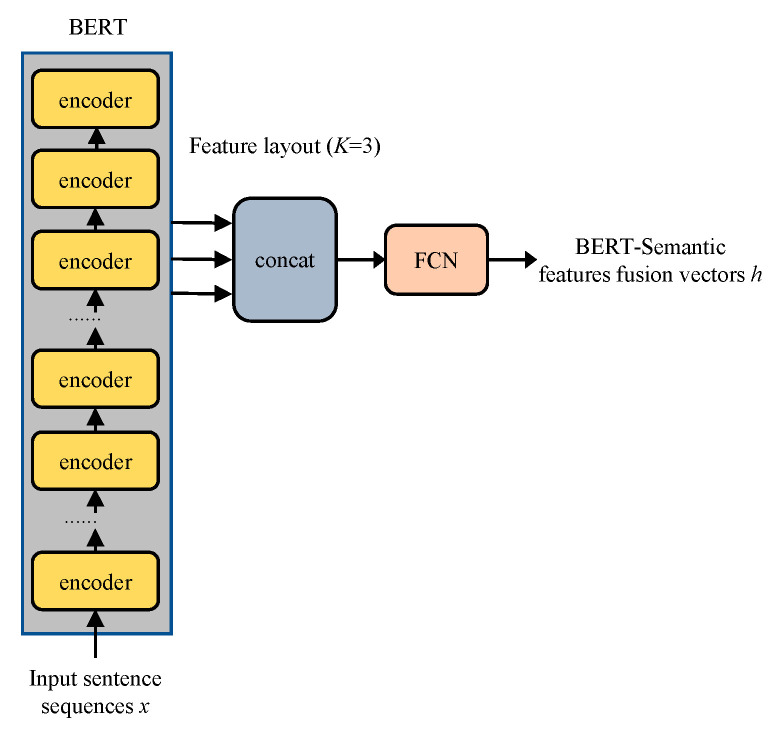
BERT-semantic fusion layer.

The JMBSF model incorporates semantic feature fusion in the construction of its semantic feature fusion layer, as demonstrated in Figure 2. The model examines the effect of semantic features by extracting the output from each layer of the BERT encoder. The output from the K-layer encoder that displays the best effect is selected, and the semantic feature vectors from these K-layers are combined into a matrix and passed through a fully connected layer to obtain the semantic features vector required by the BERT encoder in the model. Results from the subsequent ablation study indicate that the optimal results are achieved when K is set to 3, as represented in Equation (1).

(1) $$H_{\mathrm{merge}}=FC\left(\mathrm{concat}\left(H^{i},H^{j},H^{k}\right)\right)$$

where i, j, and k represent the indexes of the encoder layer of the fused BERT model.

By fusing the feature vectors from the K-layer encoder, the model obtains a fused semantic feature coding vector that fully leverages the semantic feature information extracted by BERT, resulting in better performance compared to the traditional BERT model, which uses the output from the last layer encoder as the semantic feature coding vector.

### 3.3. Bi-LSTM

This section outlines the Long Short-Term Memory (LSTM) network, a type of RNN that addresses the issue of gradient disappearance in RNNs to some extent, by introducing three types of gated network nodes, specifically, the input gate, output gate, and forgetting gate [10]. Bi-LSTM [27] is a variant of LSTM that computes in both forward and backward directions, where the forward LSTM learns the information of the current and past sequences, while the backward LSTM learns the information of the current and future sequences.

**Figure 3:**
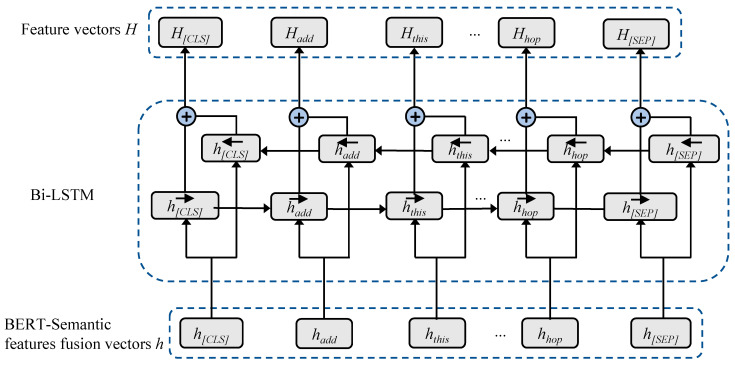
Bi-LSTM layer.

The JMBSF model Incorporates Bi-LSTM after the BERT semantic feature fusion layer, thereby enhancing the contextual encoding information, as depicted in Figure 3. Bi-LSTM obtains contextual information through the utilization of two LSTMs, and its bi-directional sequential nature allows for effective learning of the sequence location information [28], thus mitigating the problem of inadequate utilization of sequence location feature information by BERT to some extent.

### 3.4. Conditional Random Field

The CRF [29] model is a widely used approach in various sequence labeling tasks. The CRF model incorporates Markov assumptions for sequences, and its objective function takes into account both the state features of the input and the label state features. This allows the model to consider the order between output labels and make full use of the interrelationships between neighboring labels by computing the mutual probability relationships between adjacent labels and the current label [30].

The CRF imposes a conditional probability constraint on the probabilistic output of the current label, allowing the model to learn linear weights about the local information of a label in relation to its sentence. This ensures the validity of the relationship between the predicted label and its neighboring sequence labels. The integration of the CRF results in more reasonable sequence-level prediction results in sequence labeling tasks, as the model takes into account the dependencies with neighboring positions during label prediction [31].

### 3.5. Intent Classification

Intent classification is typically approached as a sentence-level multi-classification problem [32]. The pre-trained BERT model outputs a feature sequence in which the first token, [CLS], represents the overall information of the sentence. To carry out sentence-level tasks, it is conventional to input [CLS] as a global semantic feature into the downstream classifier for classification results [33], as demonstrated in Equation (2):

(2) $$\;P\left(y^{intent}\right)=softmax\left(h_{\left[\mathrm{CLS}\right]}\times W_{\mathrm{intent}}+B_{\mathrm{intent}}\right)$$

**Figure 4:**
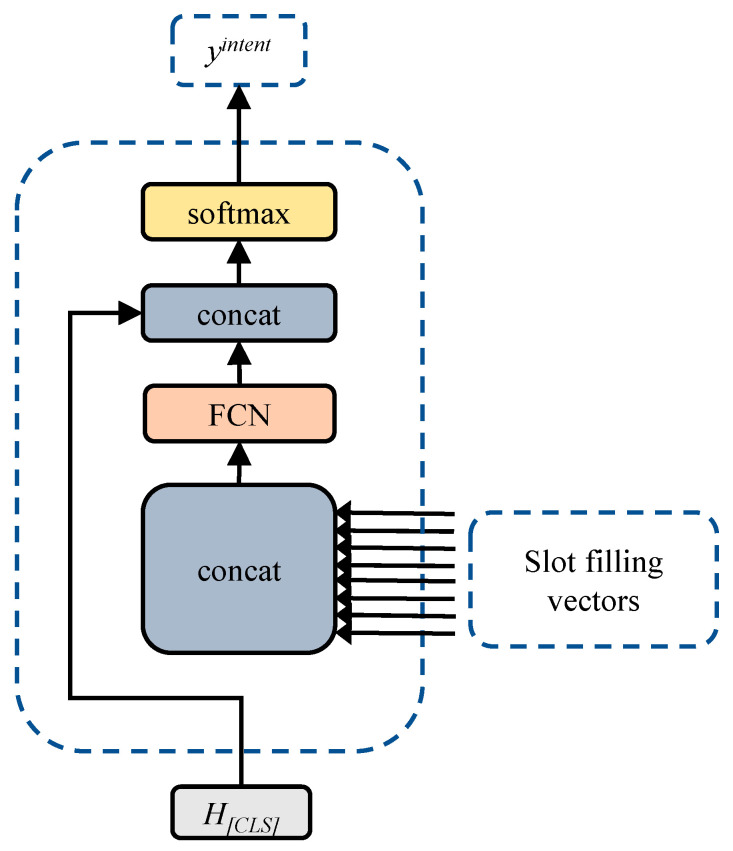
Semantic feature fusion for intent tasks.

While other tokens in the feature sequence also convey semantic features, incorporating this information into [CLS] can result in more efficient utilization of semantic features. The JMBSF model implements this concept of semantic feature fusion in Equation (2), and, as illustrated in Figure 4, the model fuses all the semantic feature vectors in the entire sentence before concatenating the resulting fused feature matrix with the [CLS] special marker vectors, as demonstrated in Equation (3).

(3) $$H_{\mathrm{merge}}=W\left[concat\left(h_{\left[CLS\right]},\left[w_{\mathrm{merge}}\times \left(concat\left(h_{1},h_{2},\ldots ,h_{n}\right)\right)+b_{merge}\right]\right)\right]+B$$

Finally, the softmax function is utilized to produce the sentence-level classification result.

(4) $$P\left(y^{intent}\right)=softmax\left(H_{merge}\times W_{intent}+B_{intent}\right)$$

Semantic fusion allows the model to make better use of the global semantic features of the whole sentence in sentence-level classification tasks, resulting in improved accuracy for intent recognition tasks.

### 3.6. Slot Filling

Slot filling is a word-level annotation task that aims to label each word in the feature sequence vector appropriately [2]. The “BIO” encoding used in this task involves dependencies between neighboring words, and thus, the JMBSF model incorporates a CRF classifier to capture these dependencies. The CRF model considers not only the current annotation but also the context of neighboring annotations [34].

The JMBSF model combines inter-layer semantic features and accesses the intent classification network to classify the intent of the sentence while simultaneously passing sentence features through the CRF network for slot filling. The joint objective function of this task is expressed as Equation (5).

(5) $$P\left(y^{i},y^{\mathrm{slots}\;|x}\right)=P\left(y^{intent}|x\right)\times \prod_{n=1}^{N}p\left(y_{n}^{\mathrm{slots}\;}|x\right)$$

where $y^{intent}$ represents the intent classification result for the fused semantic features in the sentence and $y_{n}^{\mathrm{slots}}$ is the slot-filling result for each sequence in the sentence. By optimizing the joint objective function’s probability, the model combines the probabilities of both the intent classification and slot-filling tasks, thereby improving its overall performance.

### 3.7. Datasets

In this study, two publicly accessible datasets, ATIS [21] (https://datasets.activeloop.ai/docs/ml/datasets/atis-dataset/, accessed on 26 February 2023) and Snips [22] (https://huggingface.co/datasets/snips_built_in_intents, accessed on 26 February 2023), are utilized to evaluate the performance of the JMBSF model. The ATIS dataset, which is released by the Defense Advanced Research Projects Agency, consists of 21 intent categories and 120 slot labels and is composed of 4478 training corpora, 893 validation corpora, and 500 test corpora as per the division method outlined in [2]. On the other hand, the Snips dataset, which is released by a voice assistant platform company of Snips, comprises 7 intent categories and 72 slot labels and 13,084 training corpora, 700 validation corpora, and 700 test corpora. The specific information of the dataset is shown in Table 2.

## 4. Experiments

This paper evaluates the performance of the JMBSF model using the Accuracy (Acc) and F1-score as performance metrics. Sentence accuracy is used as the evaluation index for the joint semantics of the dual task. The evaluation process includes the presentation of the evaluation metrics, training setup and procedure, and experimental results, followed by an ablation study on the added model structure of the JMBSF model to validate the model structure’s validity.

### 4.1. Evaluation Indicators

In evaluating the performance of the JMBSF model, this study employs Acc and F1-score as metrics for the model and sentence accuracy as the evaluation criterion for the joint semantics of the dual tasks. For intent classification, the accuracy rate is utilized to assess the proportion of correctly classified samples relative to the total number of samples. On the other hand, the evaluation metric for slot filling is the F1-score, which is a balanced mean of accuracy rate and recall rate. The sentence accuracy rate, in turn, represents the percentage of sentences where both intent and slot predictions of the model are exactly correct.

The Acc of the intent classification task and the F1-score of the slot-filling task are computed using the following mathematical expressions:

(6) $$\;Acc=\frac{TP+TN}{\left(TP+TN+FP+FN\right)}$$

TP (true positive) refers to the number of samples that are truly positive and are classified as positive by the model. TN (true negative) refers to the number of samples that are truly negative and are classified as negative by the model. FP (false positive) refers to the number of samples that are truly negative but are classified as positive by the model. Finally, FN (false negative) refers to the number of samples that are truly positive but are classified as negative by the model.

(7) $$F1=2*\frac{\left(precission*recall\right)}{\left(precission+recall\right)}$$

where precision is the number of true positives divided by the total number of predicted positives $\left(TP/\left(TP+FP\right)\right)$ and recall is the number of true positives divided by the total number of actual positives $\left(TP/\left(TP+FN\right)\right)$.

### 4.2. Training Setup and Procedure

In this paper, the pre-trained model BERT utilizes the BERT-base-uncased version [15], which comprises a 12-layer transformer encoder structure, 12 attention heads in the encoder multi-head attention, a hidden layer feature dimension of 768, a maximum input sequence length of 50, and an output dropout probability of 0.2. The input batch size is 32, and the training epochs are set at 20 rounds. Additionally, the hidden layer size of LSTM is set to 128, and the bi-directional output dropout probability is 0.5. The semantic feature fusion layer is designed to extract and fuse information from three semantic layers, as indicated by the choice of K = 3. The value of K is subjected to an ablation study in this paper. The model is trained using the focal loss as the loss function and the LazyAdam optimizer [20] instead of the Adam optimizer utilized in BERT.

**Figure 5:**
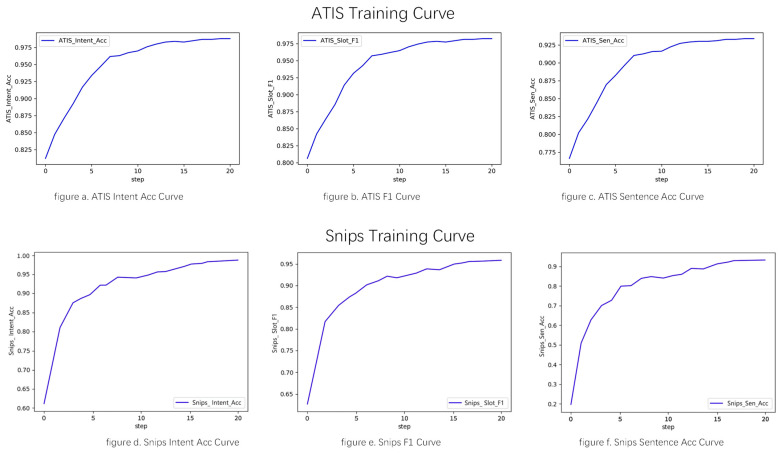
Training curves for accuracy, F1, and sentence accuracy on ATIS and Snips datasets.

Our model is evaluated in an NVIDIA GeForce RTX 3070 8G GPU with CUDA version 11.6, Python version 3.8.3, and PyTorch version 1.8.0. During training, the model is trained for a total of 20 epochs, and the resulting curves for accuracy, F1 value, and sentence accuracy are plotted against the epochs in Figure 5. Upon completion of the training phase, the model’s inference time measures between 12–16 ms using the same experimental environment.

### 4.3. Results and Analysis

The comparison of the experimental results of the JMBSF model with other joint models is presented in Table 3. The results demonstrate an improvement in the performance of the JMBSF model, both in terms of single-task intent recognition and slot filling and in terms of overall classification accuracy.

In this paper, the proposed JMBSF model is compared with other joint models, including Atten-BiRNN [10], Joint seq [27], Slot-gated [35], Joint BERT [2], BiAss-Gate [13], Stack-propagation BERT [17], and Typed abstraction mechanism BERT [18]. Atten-BiRNN employs a bi-directional RNN for feature extraction, while Joint seq integrates RNN-LSTM architecture for the joint modeling of slot-filling and intent classifications. The Slot-gated model also utilizes a gating mechanism to guide the slot-filling task, but its limited access to intent embedding representation information results in subpar model performance. The Joint BERT model was the first to utilize a pre-trained model for feature extraction and achieved superior results by employing the BERT and CRF architecture. The BiAss-Gated model improves upon the Slot-gated model by incorporating CRF after the Bi-LSTM structure, modeling the relationship between intent and semantic slots using intent and semantic slot context vectors, and incorporating gated networks to enhance overall classification accuracy. Stack-propagation BERT incorporating intent information into slot filling through a joint model with Stack-Propagation and token-level intent classification is proposed to capture intent semantic knowledge and reduce error propagation. Typed abstraction mechanism BERT presents a novel approach to improve intent classification by utilizing encoded information from semantic feature tasks and mitigating error propagation through a typed iteration approach.

In comparison, the JMBSF model demonstrates improved performance in both the ATIS [21] and Snips [22] datasets, surpassing the results of the Joint BERT and Typed abstraction mechanism BERT and achieving state-of-the-art performance in two public datasets. In the ATIS dataset, the intent classification accuracy, slot-filling F1-score, and sentence accuracy of the JMBSF model are 98.80%, 98.25%, and 93.40%, respectively, representing improvements of 1.25%, 2.53%, and 5.99% compared to the Joint BERT model [2] and improvements of 0.71%, 2.13%, and 5.30% compared to the Typed abstraction mechanism BERT [18]. Similarly, in the Snips dataset, the intent classification accuracy, slot-filling F1-score, and sentence accuracy of the JMBSF model are 99.71%, 97.24%, and 93.57%, respectively, compared to the Joint BERT model [2] with improvements of 0.95%, 1.10%, and 2.95%, and compared to the Typed abstraction mechanism BERT [18] with improvements of 0.82%, 0.56%, and 1.49%. These results suggest that the JMBSF model, based on BERT and semantic fusion, is capable of effectively exploiting semantic feature information to enhance SLU performance compared to previous joint models. Among the three evaluation metrics, sentence accuracy, which reflects the joint impact of the model on both tasks, demonstrates significant improvement, indicating that the semantic feature fusion approach of the JMBSF model can effectively utilize sentence contextual semantic features to enhance the joint model’s results, optimize the use of semantic features across layers, and improve the overall performance of the model for both tasks.

### 4.4. Ablation Experiment

The results of the ablation study in this paper demonstrate the validity of the model structures proposed. This study uses the BERT + CRF model as a baseline and evaluates it using the ATIS [21] and Snips [22] datasets, as presented in Table 4. The results show that incorporating a Bi-LSTM structure in the BERT + CRF model leads to improvement across various metrics, suggesting that the model can effectively learn contextual and location information. This study also examines the impact of introducing a feature fusion layer in the intent classification task, and the results from Table 4 indicate that it can improve intent classification accuracy and sentence accuracy to some extent but has less impact on slot-filling accuracy. The results further reveal that the incorporation of the feature fusion layer in both the feature extraction and intent classification stages leads to significant improvements in all three metrics, indicating that it can effectively learn contextual and feature information in the feature extraction stage.

Additionally, the ablation study investigates the impact of varying the number of layers in the feature fusion layer, with the results presented in Table 5. As the number of layers K increases from 1 to 3, the evaluation metrics gradually improve, demonstrating that the feature fusion layer can effectively fuse more feature information and learn more semantic features. However, when K exceeds 4 and 5, the improvement in the intent classification task reaches a plateau, and the word-level slot-filling performance worsens, leading to a decline in the overall sentence accuracy. Based on these findings, this paper selects K=3 as the number of layers in the feature fusion layer.

## 5. Conclusions

This paper presents the JMBSF to tackle the issues of inadequate correlation of contextual information and underutilization of semantic feature information in intent classification and semantic slot-filling deep learning models. The JMBSF model employs a pre-trained BERT as a semantic feature extractor to enhance the generalization capability and utilizes feature fusion to combine the semantic feature vectors from the hidden layer of the BERT multi-layer encoder, thereby allowing for the association with context and maximizing the utilization of semantic feature information. The addition of a Bi-LSTM network further facilitates the learning of contextual location information. Furthermore, semantic features are once again fused in the intent classification process, and a CRF model is utilized for classification in slot filling, thereby effectively learning label pre-post dependencies and improving model performance. The training efficiency is optimized by utilizing the focal loss instead of cross-entropy loss as the loss function for intent classification and LazyAdam instead of Adam optimizer in BERT. The proposed JMBSF model exhibits substantial improvement compared to other joint models on the ATIS and Snips datasets, especially in sentence accuracy. Future work includes compressing the model through techniques, such as model distillation or pruning, to reduce the number of model parameters, thereby improving inference speed and exploring multi-intent classification to enhance the effectiveness of SLU tasks in complex sentences.

## Figures and Tables

**Table 1:** Sample example.

Sample	Add	This	Track	To	New	Hip	Hop
Slot	O	O	B-music_item	O	B-playlist	I-playlist	I-playlist
Intent	AddToPlaylist						

**Table 2:** Specific statistics of the two datasets.

Dataset	ATIS	SNIPS
Vocabulary size	722	11,241
Intents	21	7
Slots	120	72
Training samples	4478	13,084
Validation samples	500	700

**Table 3:** Performance comparison of different joint models on ATIS and Snips datasets %.

Models	ATIS			Snips		
	Intent Acc	Slot F1	Sen Acc	Intent Acc	Slot F1	Sen Acc
Atten-BiRNN [10]	91.10	94.20	78.90	96.70	87.80	74.10
Joint seq [27]	92.60	94.30	80.70	96.90	87.30	73.20
Slot-gated [35]	94.10	95.20	82.60	97.00	88.80	75.50
BiAss-Gate [13]	97.09	95.80	86.56	98.29	93.62	84.43
Joint BERT [2]	97.58	95.83	88.12	98.77	96.18	90.89
Stack-propagation + BERT [17]	97.30	96.10	88.20	98.60	96.70	91.80
Typed abstraction mechanism + BERT [18]	98.10	96.20	88.70	98.90	96.70	92.20
JMBSF	**98.80**	**98.25**	**93.40**	**99.71**	**97.24**	**93.57**

**Table 4:** Comparison of ablated model structures between ATIS and Snips datasets %.

Models	ATIS			Snips		
	Intent Acc	Slot F1	Sen Acc	Intent Acc	Slot F1	Sen Acc
BERT + CRF	97.40	97.87	90.60	98.57	95.50	89.43
BERT + CRF + Bi-LSTM	97.62	98,12	91.2	98.77	96.30	90.29
BERT + CRF + merge_layer *	97.80	97.72	91.8	98.83	95.78	90.71
JMBSF *	98.00	98.16	92.00	99.11	96.52	92.14
JMBSF	**98.80**	**98.25**	**93.40**	**99.71**	**97.24**	**93.57**

* (only for intent).

**Table 5:** Comparison of K-value ablation studies in feature fusion %.

JMBSF	ATIS			Snips		
	Intent Acc	Slot F1	Sen Acc	Intent Acc	Slot F1	Sen Acc
K = 1	98.00	98.16	92.00	99.11	96.52	92.14
K = 2	98.32	98.19	93.12	99.54	96.93	92.74
K = 3	98.80	98.25	93.40	99.71	97.24	93.57
K = 4	98.85	98.09	93.22	99.80	97.07	93.28
K = 5	98.89	97.93	93.06	99.83	96.96	93.13

## Data Availability

Not applicable.
